# Neurosurgical innovation: balancing the risks and benefits of technical novelty

**DOI:** 10.3171/2019.10.FocusVid.Editorial2

**Published:** 2019-10-01

**Authors:** Aaron A. Cohen-Gadol

**Affiliations:** Associate Editor-in-Chief, *Neurosurgical Focus: Video*; Department of Neurosurgery, Indiana University, Indianapolis, Indiana

I commend Drs. Gardner, Snyderman, and colleagues for their pioneering work to advance the field of endoscopic skull base surgery. Their surgical skills have demonstrated the prospects of operating via endoscopic endonasal techniques.

Gardner and Snyderman’s “Endoscopic Endonasal Approach for Brainstem Cavernous Malformation” video in the current issue of *Neurosurgical Focus: Video* clearly demonstrates that the boundaries of the endonasal route for reaching intraaxial lesions within highly critical regions can be pushed farther. Although I enjoyed watching this video and might attempt using this operative corridor in a similar case in the future, I could not resist considering the alternative operative routes for reaching this lesion. Naturally, the dilemma for the innovative surgeon is how to balance the risks and uncertainties of using a completely novel operative corridor against those of using a more established surgical pathway. For the patient in this video, I might have considered using the transcallosal route, because the malformation will be readily visible at the wall of the third ventricle. This route would have traversed less highly functional nervous tissue, avoided the precious basilar artery perforators, and minimized the risk of a cerebrospinal fluid fistula.

However, the transventricular route to the third ventricle is not without significant risks to the critical periventricular structures, including the fornices. Interhemispheric dissection of the cinguli and partial callosotomy accompany their own set of risks. Subtle neuropsychological deficits associated with these dissections are often acceptable by the surgeon but not by the patient.

The art of balancing the risks of these different routes defines surgical experience and “intuition.” One has to honestly gauge his or her technical skills and place the interest of the patient as the highest priority. However, without surgeons taking risks, the introduction of novel operative approaches would be unlikely. Innovative operators have years of experience and, most importantly, a selfless passion to strive for technical excellence and an ability to candidly self-reflect and respond constructively to criticism.

I want to again congratulate the authors for pushing the boundaries of the field via their immense expertise.

